# Supplementary material for: Identifying bias in models that detect vocal fold paralysis from audio recordings using explainable machine learning and clinician ratings
Source: PLOS Digit Health. 2024 May 30;3(5):e0000516. doi: 10.1371/journal.pdig.0000516 (PMC11139298; doi:10.1371/journal.pdig.0000516)
Supplement: S1 Appendix — Text A. List of eGeMAPs features. Figure A. Controls, reading+vowel tasks: Visualization of features with shared information using pairwise distance correlation across the 88 features. Squares are clusters of redundant features. Figure B. Non-monotonic associations between features. Figure C. All participants, reading task: Visualization of features with shared information using pairwise distance correlation across the 88 eGeMAPs features. Squares are clusters of redundant features. Figure D. All participants, vowel task: Visualization of features with shared information using pairwise distance correlation across the 88 eGeMAPs features. Squares are clusters of redundant features. Figure E. All participants, reading+vowel tasks: Visualization of features with shared information using pairwise distance correlation across the 88 features. Squares are clusters of redundant features. Figure F. Patients, reading task: Visualization of features with shared information using pairwise distance correlation across the 88 eGeMAPs features. Squares are clusters of redundant features. Figure G. Patients, vowel task: Visualization of features with shared information using pairwise distance correlation across the 88 eGeMAPs features. Squares are clusters of redundant features. Figure H. Patients, reading+vowel tasks: Visualization of features with shared information using pairwise distance correlation across the 88 eGeMAPs features. Squares are clusters of redundant features. Figure I. Controls, reading task: Visualization of features with shared information using pairwise distance correlation across the 88 eGeMAPs features. Squares are clusters of redundant features. Figure J. Controls, vowel task: Visualization of features with shared information using pairwise distance correlation across the 88 eGeMAPs features extracted. Squares are clusters of redundant features. Figure K. Controls, reading+vowel tasks: Visualization of features with shared information using pairwise distance [file pdig.0000516.s001.pdf]

Supplementary Materials

Identifying bias in models that detect vocal fold paralysis from audio recordings using explainable machine learning and clinician ratings

Daniel M. Low [1,2], Vishwanatha Rao [3,4], Gregory Randolph [4,5], Phillip C. Song [4,5] *, Satrajit S. Ghosh, PhD[1,2,5] *

[1] Program in Speech and Hearing Bioscience and Technology, Harvard Medical School, Boston, MA, USA

[2] McGovern Institute for Brain Research, MIT, Cambridge, MA, USA

[3] Department of Biomedical Engineering, Columbia University, New York, NY, USA

[4] Department of Otolaryngology–Head and Neck Surgery, Massachusetts Eye and Ear Infirmary, Boston, MA, USA

[5] Department of Otolaryngology–Head and Neck Surgery, Harvard Medical School, Boston, MA, USA

* Equal contribution

Corresponding author

Correspondence can be addressed to Daniel M. Low, Office: 46-4033F, 43 Vassar St, Cambridge, MA 02139, USA. E-mail: dlow@mit.edu.

METHODS

Text A

List of eGeMAPs features

Here is a list of features used from eGeMAPS (see main manuscript for citation and source code):
F0semitoneFrom27.5Hz_sma3nz_amean,
F0semitoneFrom27.5Hz_sma3nz_meanFallingSlope,
F0semitoneFrom27.5Hz_sma3nz_meanRisingSlope,
F0semitoneFrom27.5Hz_sma3nz_pctlrange0-2,
F0semitoneFrom27.5Hz_sma3nz_percentile20.0,
F0semitoneFrom27.5Hz_sma3nz_percentile50.0,
F0semitoneFrom27.5Hz_sma3nz_percentile80.0,
F0semitoneFrom27.5Hz_sma3nz_stddevFallingSlope,
F0semitoneFrom27.5Hz_sma3nz_stddevNorm,
F0semitoneFrom27.5Hz_sma3nz_stddevRisingSlope, F1amplitudeLogRelF0_sma3nz_amean,
F1amplitudeLogRelF0_sma3nz_stddevNorm, F1bandwidth_sma3nz_amean,
F1bandwidth_sma3nz_stddevNorm, F1frequency_sma3nz_amean,
F1frequency_sma3nz_stddevNorm, F2amplitudeLogRelF0_sma3nz_amean,
F2amplitudeLogRelF0_sma3nz_stddevNorm, F2bandwidth_sma3nz_amean,
F2bandwidth_sma3nz_stddevNorm, F2frequency_sma3nz_amean,
F2frequency_sma3nz_stddevNorm, F3amplitudeLogRelF0_sma3nz_amean,
F3amplitudeLogRelF0_sma3nz_stddevNorm, F3bandwidth_sma3nz_amean,
F3bandwidth_sma3nz_stddevNorm, F3frequency_sma3nz_amean,
F3frequency_sma3nz_stddevNorm, HNRdBACF_sma3nz_amean,
HNRdBACF_sma3nz_stddevNorm, MeanUnvoicedSegmentLength,
MeanVoicedSegmentLengthSec, StddevUnvoicedSegmentLength,
StddevVoicedSegmentLengthSec, VoicedSegmentsPerSec, alphaRatioUV_sma3nz_amean,
alphaRatioV_sma3nz_amean, alphaRatioV_sma3nz_stddevNorm, equivalentSoundLevel_dBp,
hammarbergIndexUV_sma3nz_amean, hammarbergIndexV_sma3nz_amean,
hammarbergIndexV_sma3nz_stddevNorm, jitterLocal_sma3nz_amean,
jitterLocal_sma3nz_stddevNorm, logRelF0-H1-A3_sma3nz_amean,
logRelF0-H1-A3_sma3nz_stddevNorm, logRelF0-H1-H2_sma3nz_amean,
logRelF0-H1-H2_sma3nz_stddevNorm, loudnessPeaksPerSec, loudness_sma3_amean,
loudness_sma3_meanFallingSlope, loudness_sma3_meanRisingSlope,
loudness_sma3_pctlrange0-2, loudness_sma3_percentile20.0, loudness_sma3_percentile50.0,
loudness_sma3_percentile80.0, loudness_sma3_stddevFallingSlope,
loudness_sma3_stddevNorm, loudness_sma3_stddevRisingSlope, mfcc1V_sma3nz_amean,
mfcc1V_sma3nz_stddevNorm, mfcc1_sma3_amean, mfcc1_sma3_stddevNorm,

mfcc2V_sma3nz_amean, mfcc2V_sma3nz_stddevNorm, mfcc2_sma3_amean, mfcc2_sma3_stddevNorm, mfcc3V_sma3nz_amean, mfcc3V_sma3nz_stddevNorm, mfcc3_sma3_amean, mfcc3_sma3_stddevNorm, mfcc4V_sma3nz_amean, mfcc4V_sma3nz_stddevNorm, mfcc4_sma3_amean, mfcc4_sma3_stddevNorm, shimmerLocaldB_sma3nz_amean, shimmerLocaldB_sma3nz_stddevNorm, slopeUV0-500_sma3nz_amean, slopeUV500-1500_sma3nz_amean, slopeV0-500_sma3nz_amean, slopeV0-500_sma3nz_stddevNorm, slopeV500-1500_sma3nz_amean, slopeV500-1500_sma3nz_stddevNorm, spectralFluxUV_sma3nz_amean, spectralFluxV_sma3nz_amean, spectralFluxV_sma3nz_stddevNorm, spectralFlux_sma3_amean, spectralFlux_sma3_stddevNorm.

Choosing amount of bootstrapping splits

We chose the default of 50 bootstrapping splits from pydra-ml. Additional testing shows median ROC AUC stabilized to larger spit values at around 40 splits for logistic regression models across tasks for using 88 features (see Figure A).

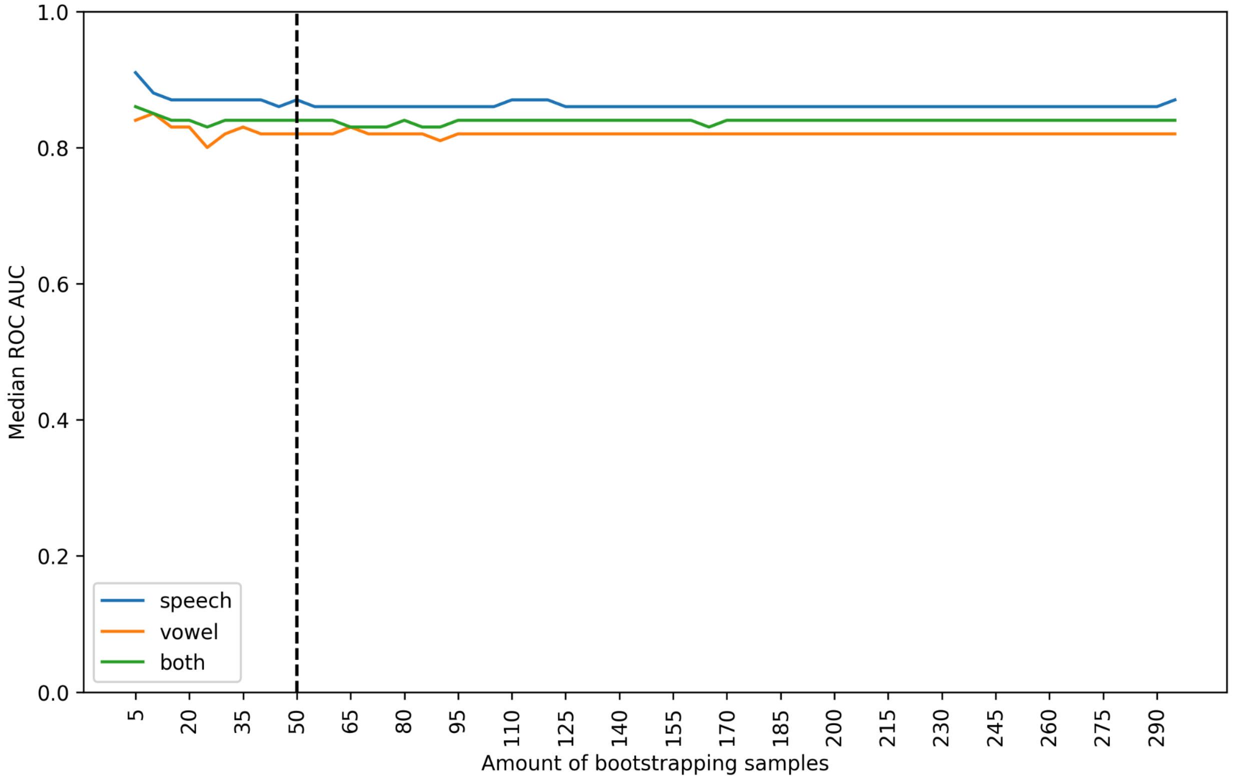

Figure A. Controls, reading+vowel tasks: Visualization of features with shared information using pairwise distance correlation across the 88 features. Squares are clusters of redundant features.

Non-monotonic association captured better by distance correlation

See Figure B for non-monotonic associations. We highlight the trend using generalized additive models from R's ggplot2 geom_smooth() function.

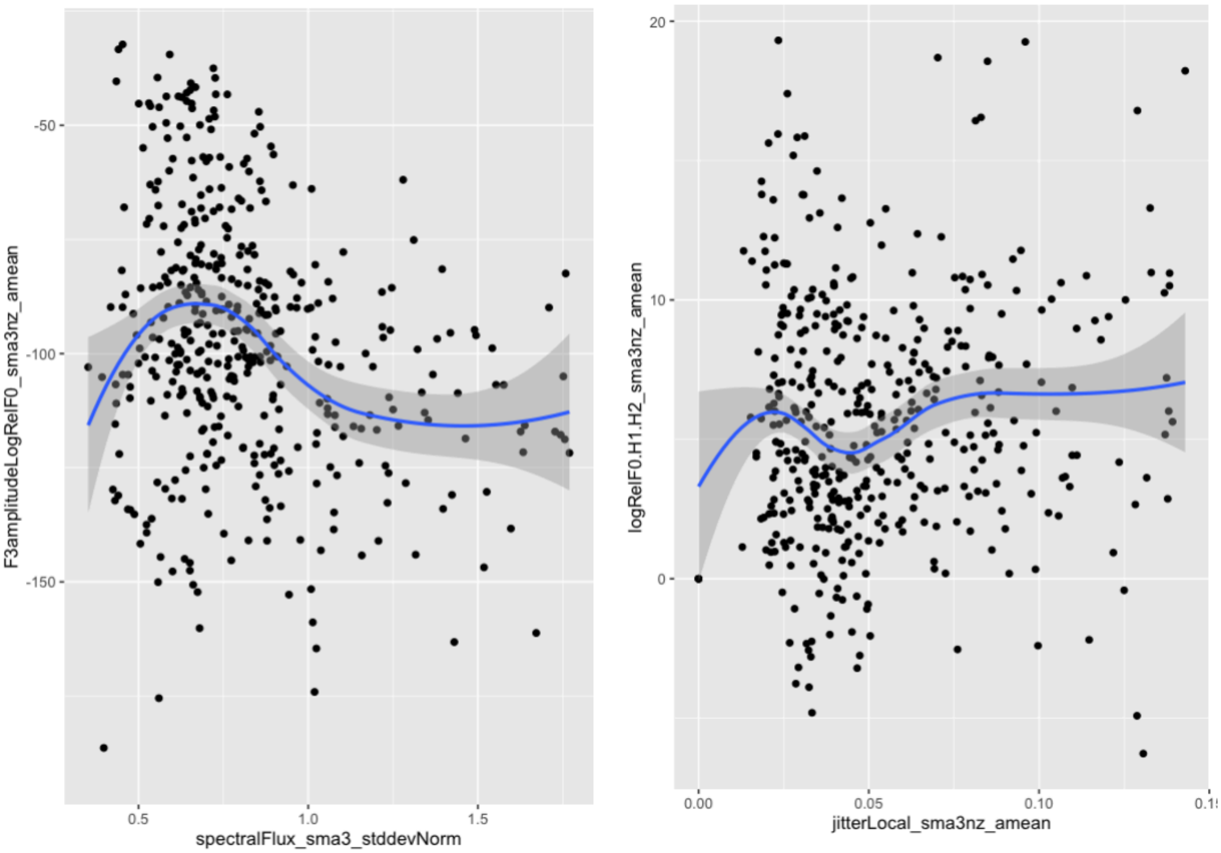

Figure B. Non-monotonic associations between features.

RESULTS

Performance using hyperparameter tuning

	Features	LogisticRegression	MLP	RandomForest	SGDClassifier
Reading	88	.85 (.78–.93)	.87 (.8–.92)	.86 (.77–.91)	.86 (.75–.92)
Vowel	88	.86 (.77–.92)	.84 (.74–.92)	.84 (.77–.93)	.86 (.78–.91)
Reading+Vowel	88	.84 (.77–.90)	.86 (.78–.90)	.86 (.80–.92)	.83 (.76–.90)

Table A. Model performance after hyperparameter tuning increased performance by 0.01 on average across models and tasks.

Visualization of Redundant Features

See Figure C–K for a visualization of redundant features for all participants, patients, and controls and for reading, vowel, and reading+vowel tasks. Top 5 features are highlighted in bold and their rank is displayed before the feature name with the corresponding leaf marked with an "x". When stratifying samples by disorder and task, clustering becomes more homogenous (clusters tend to contain a single feature type) in comparison to when all participants or both tasks are included as in Figure E. Even in Figure E, the chosen color-coded classification of features appears to be empirically replicated in this dataset given most low-level clusters (i.e., have higher dependency) are for the most part homogenous (i.e., of the same color). This also allows us to observe exceptions (e.g., mean spectral flux clusters with loudness features) which could otherwise be missed if using only a priori theoretical knowledge.

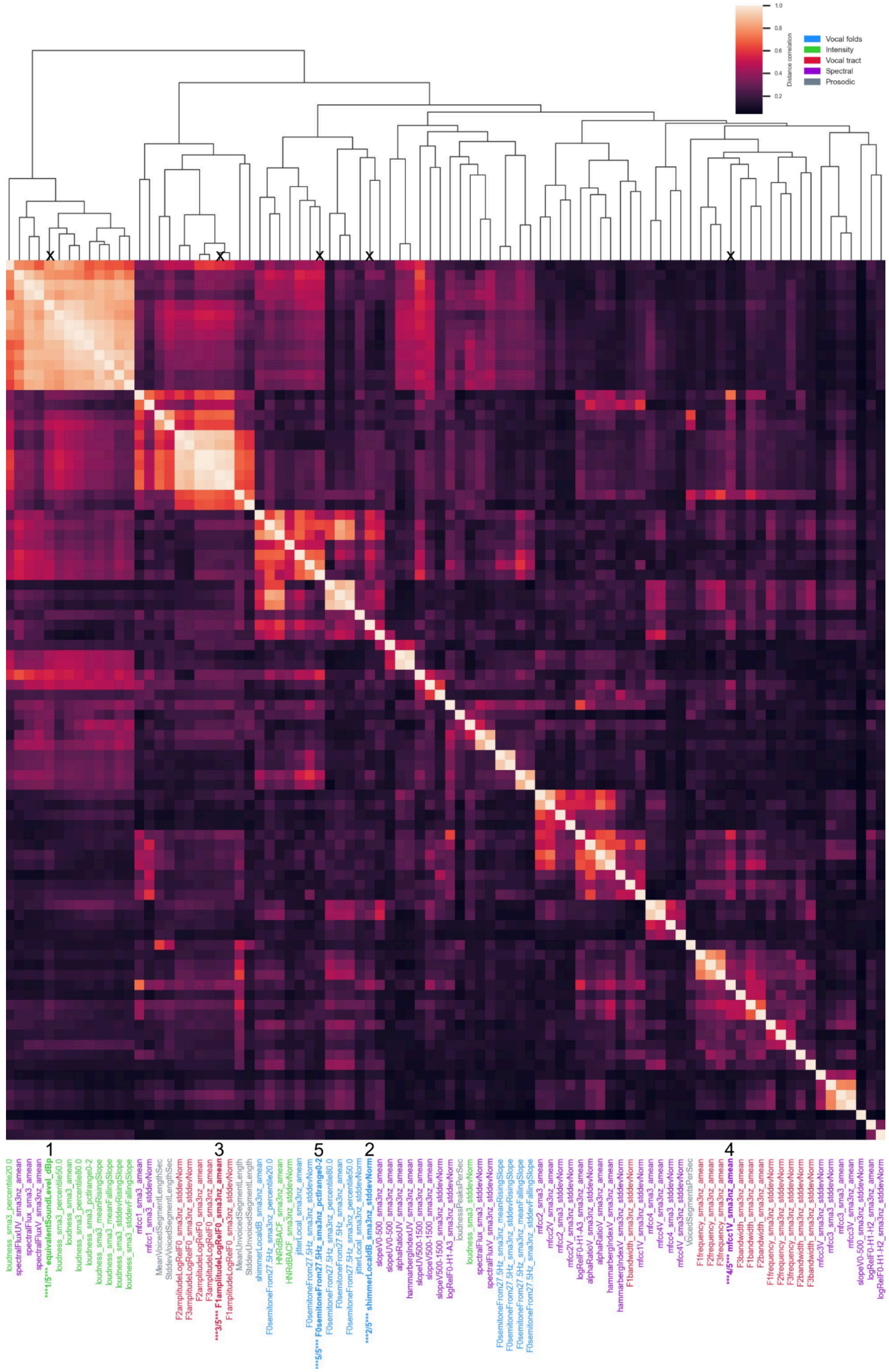

Figure C. All participants, reading task: Visualization of features with shared information using pairwise distance correlation across the 88 eGeMAPs features. Squares are clusters of redundant features.

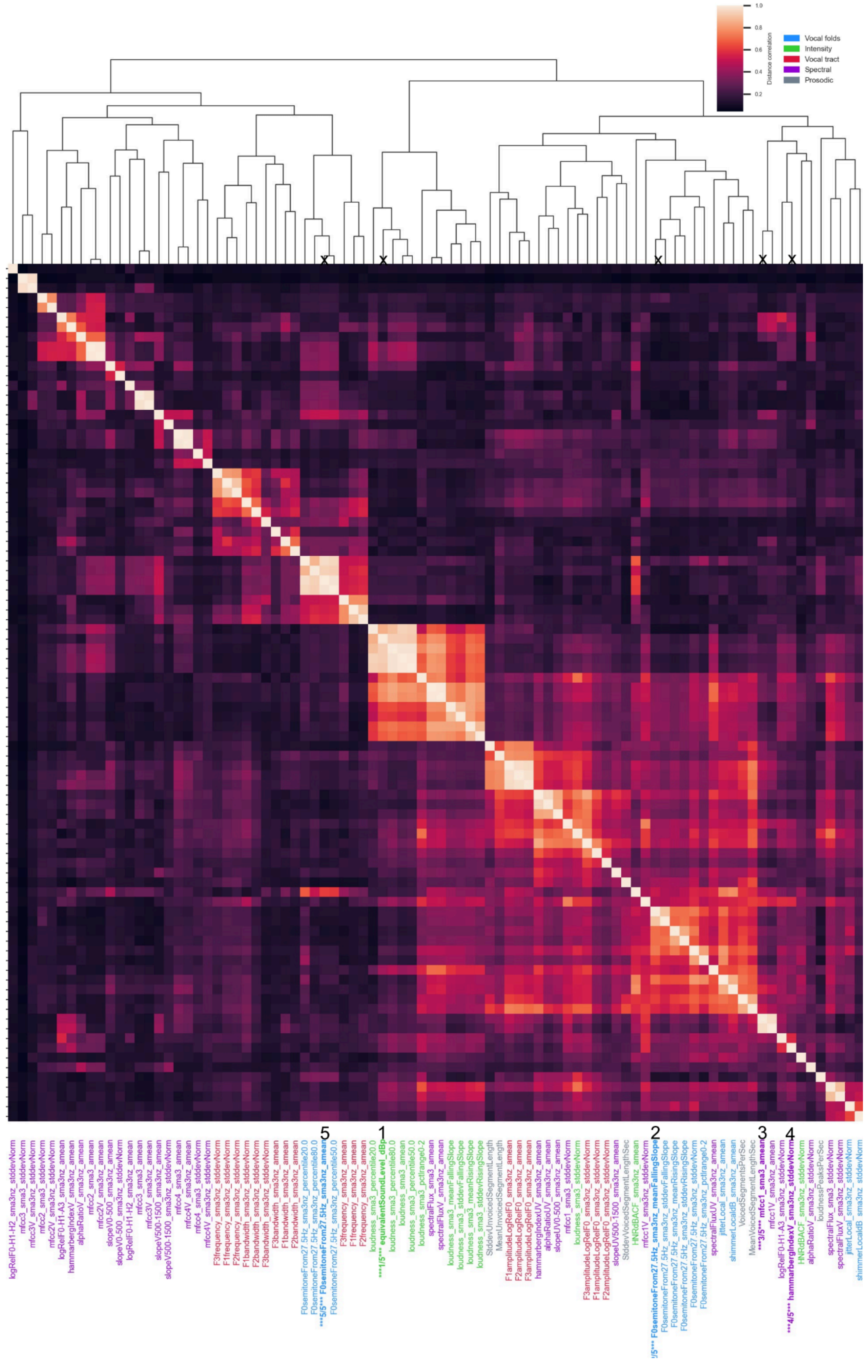

Figure D. All participants, vowel task: Visualization of features with shared information using pairwise distance correlation across the 88 eGeMAPs features. Squares are clusters of redundant features.

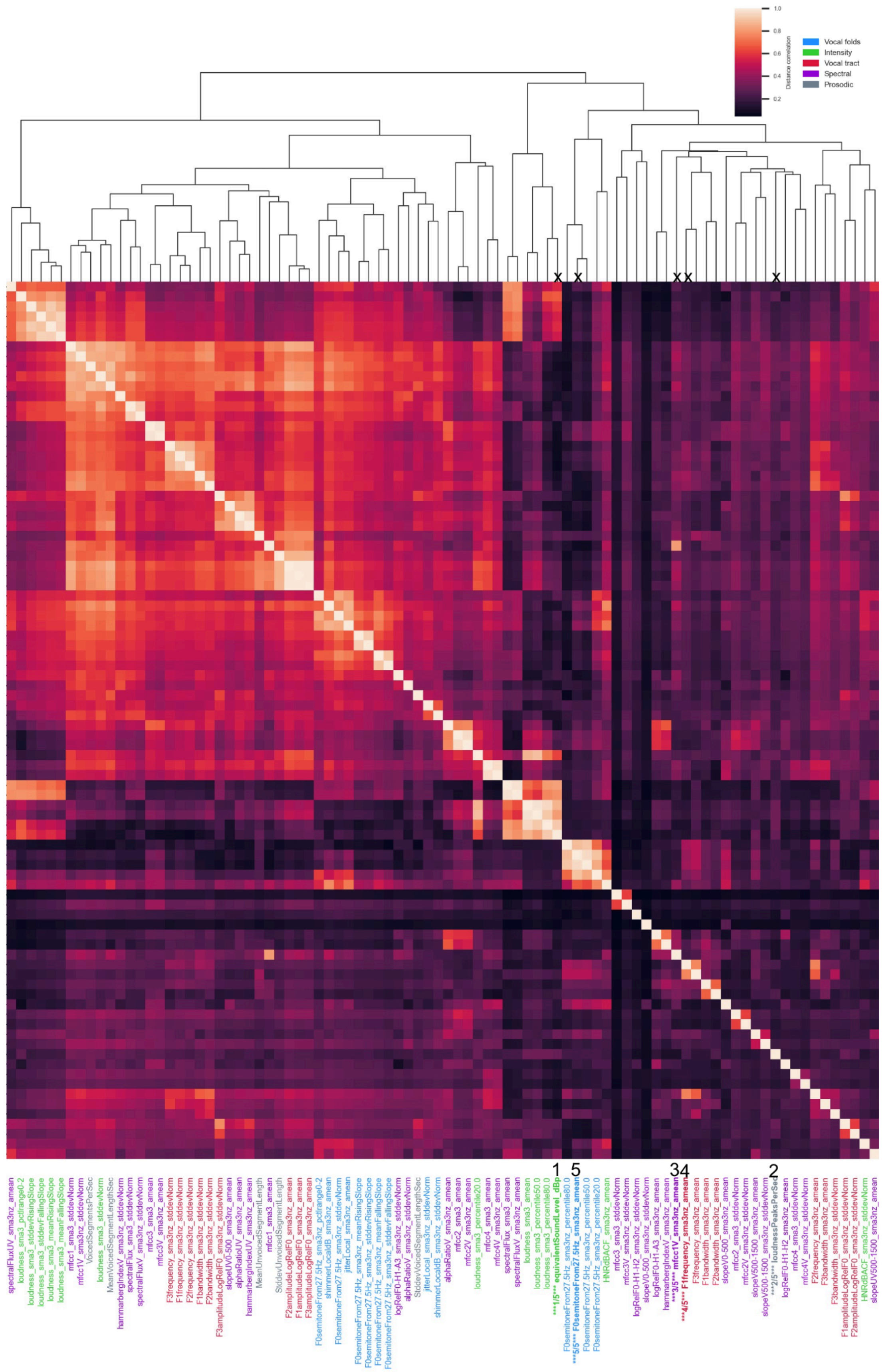

Figure E. All participants, reading+vowel tasks: Visualization of features with shared information using pairwise distance correlation across the 88 features. Squares are clusters of redundant features.

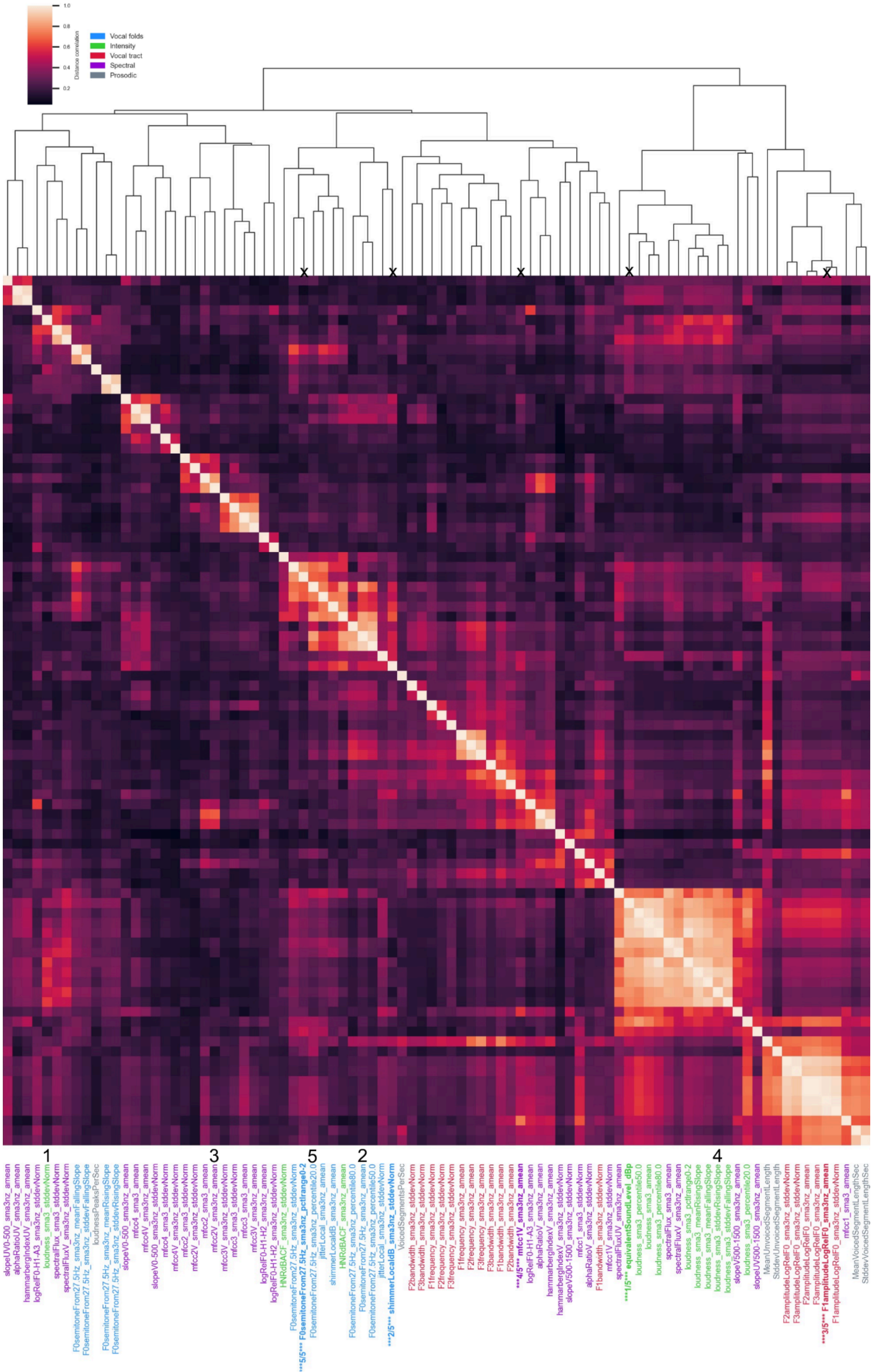

Figure F. Patients, reading task: Visualization of features with shared information using pairwise distance correlation across the 88 eGeMAPs features. Squares are clusters of redundant features.

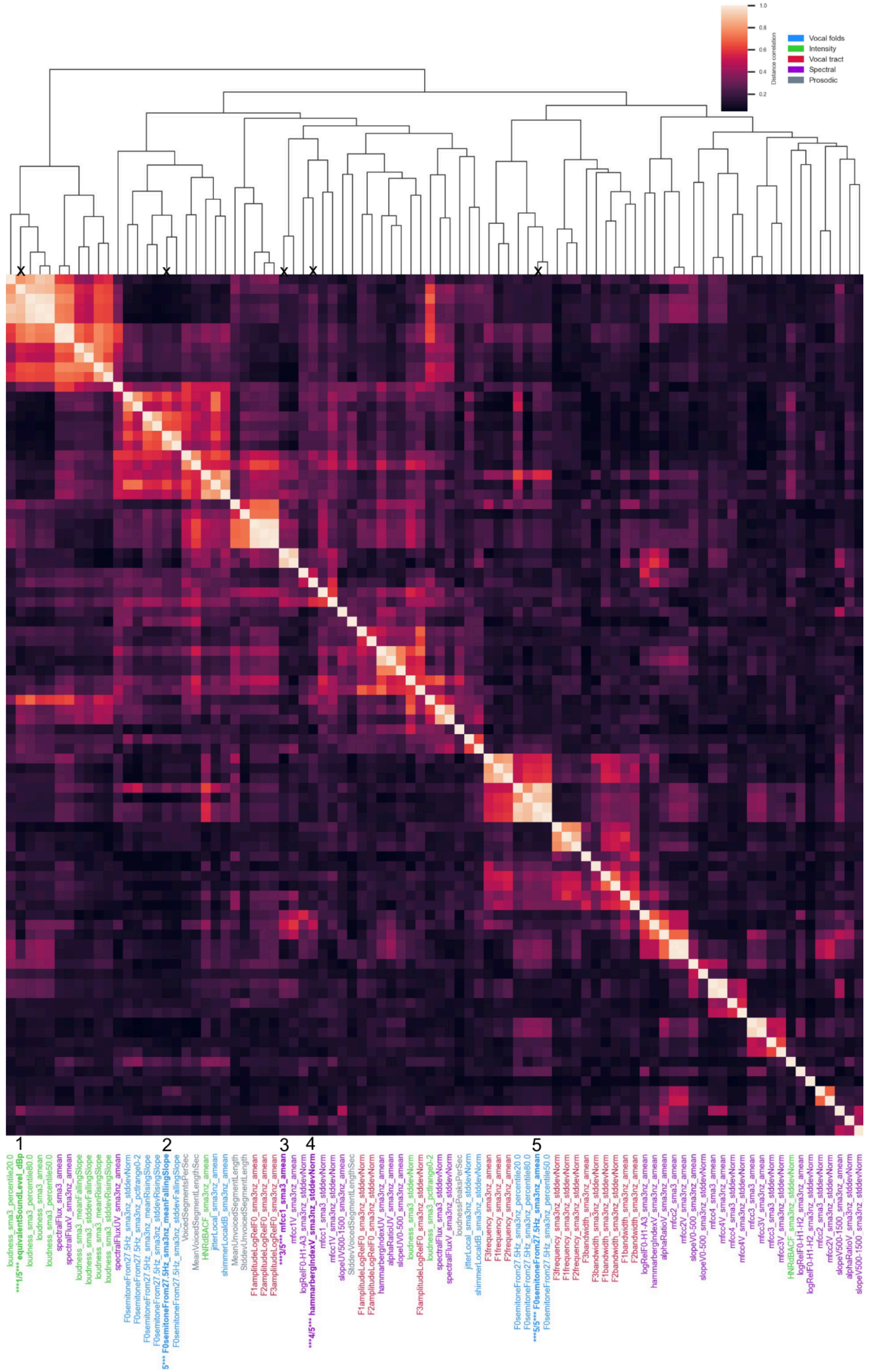

Figure G. Patients, vowel task: Visualization of features with shared information using pairwise distance correlation across the 88 eGeMAPs features. Squares are clusters of redundant features.

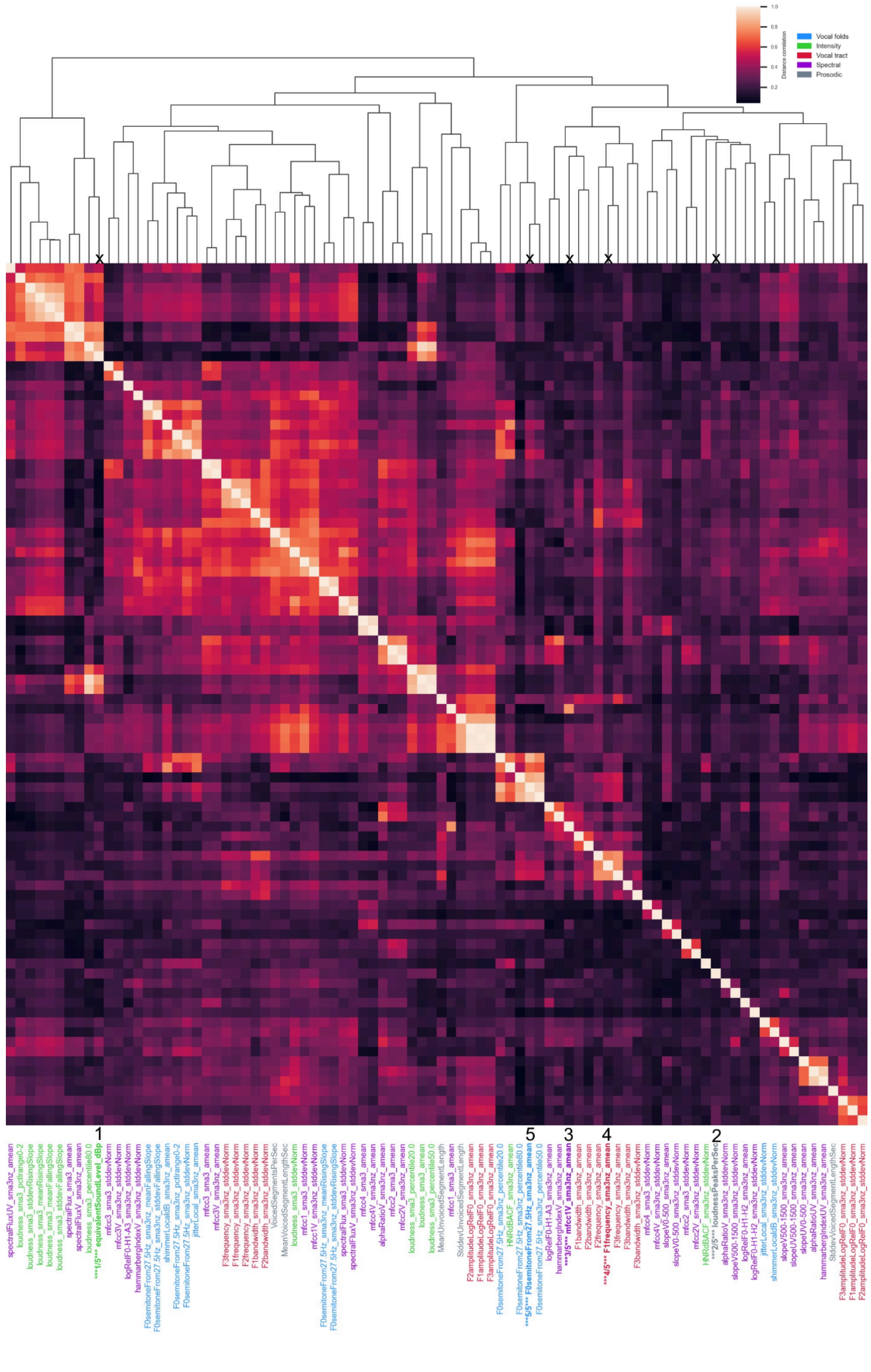

Figure H. Patients, reading+vowel tasks: Visualization of features with shared information using pairwise distance correlation across the 88 eGeMAPs features. Squares are clusters of redundant features.

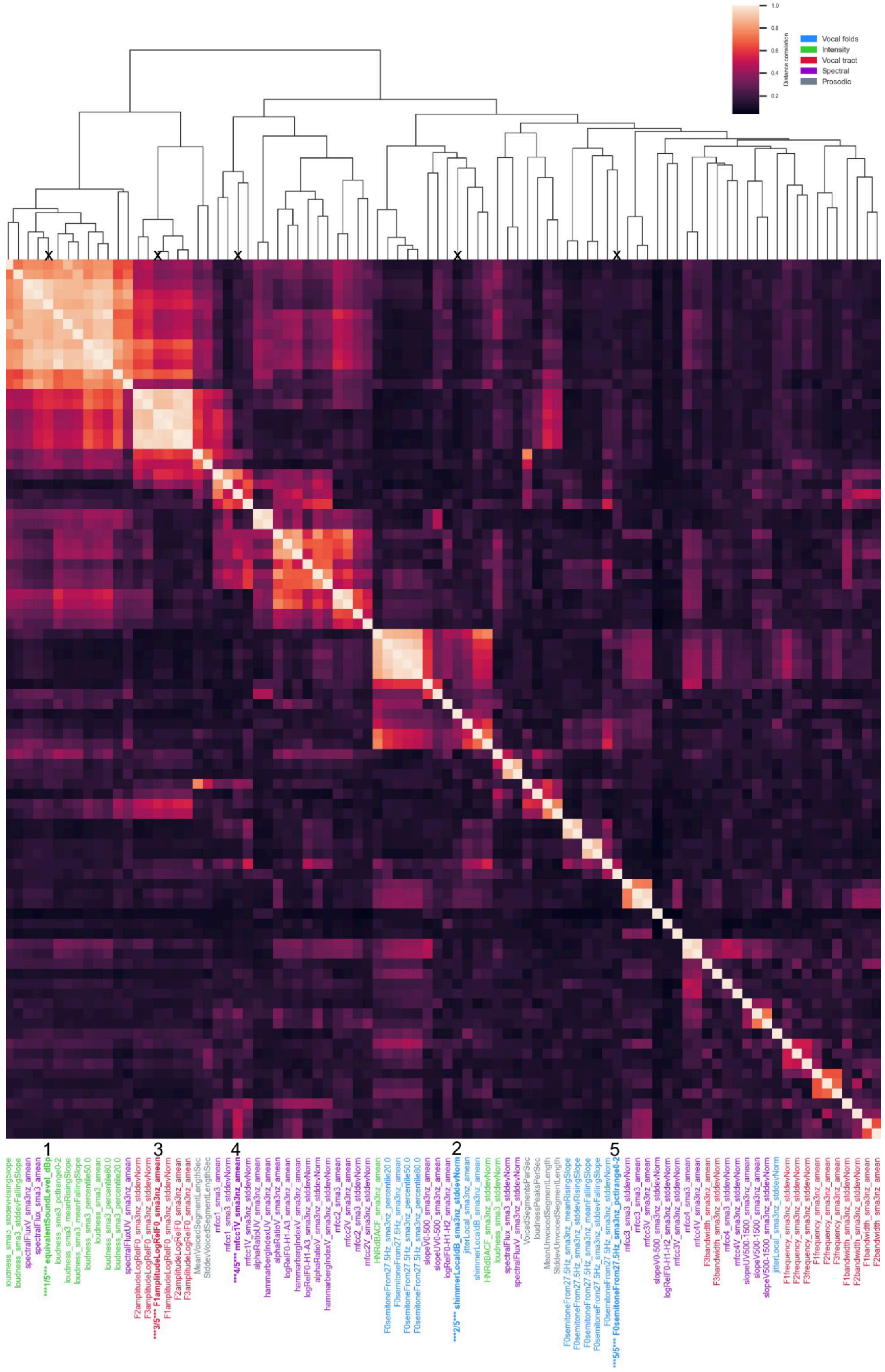

Figure I. Controls, reading task: Visualization of features with shared information using pairwise distance correlation across the 88 eGeMAPs features. Squares are clusters of redundant features.

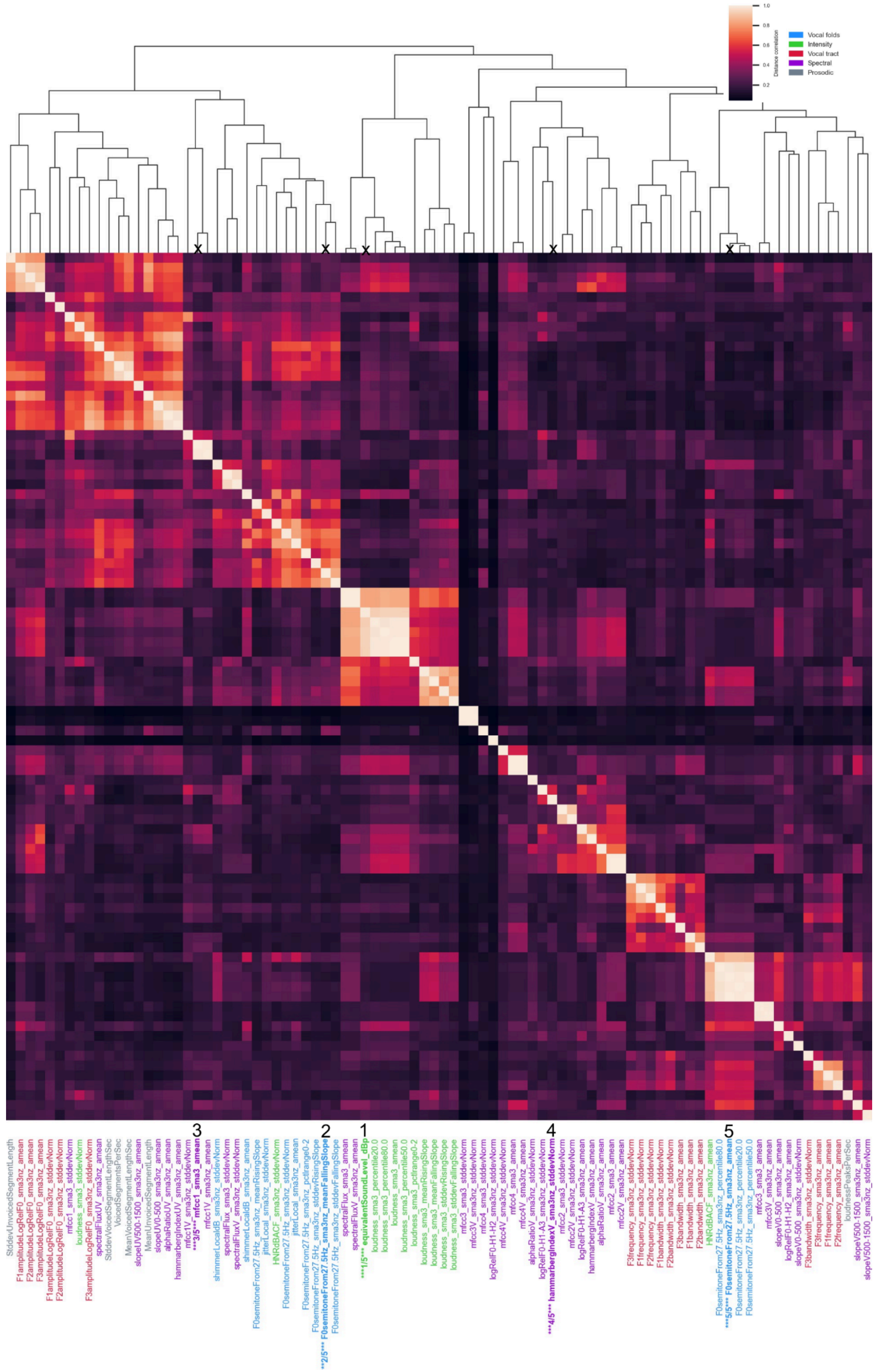

Figure J. Controls, vowel task: Visualization of features with shared information using pairwise distance correlation across the 88 eGeMAPs features extracted. Squares are clusters of redundant features.

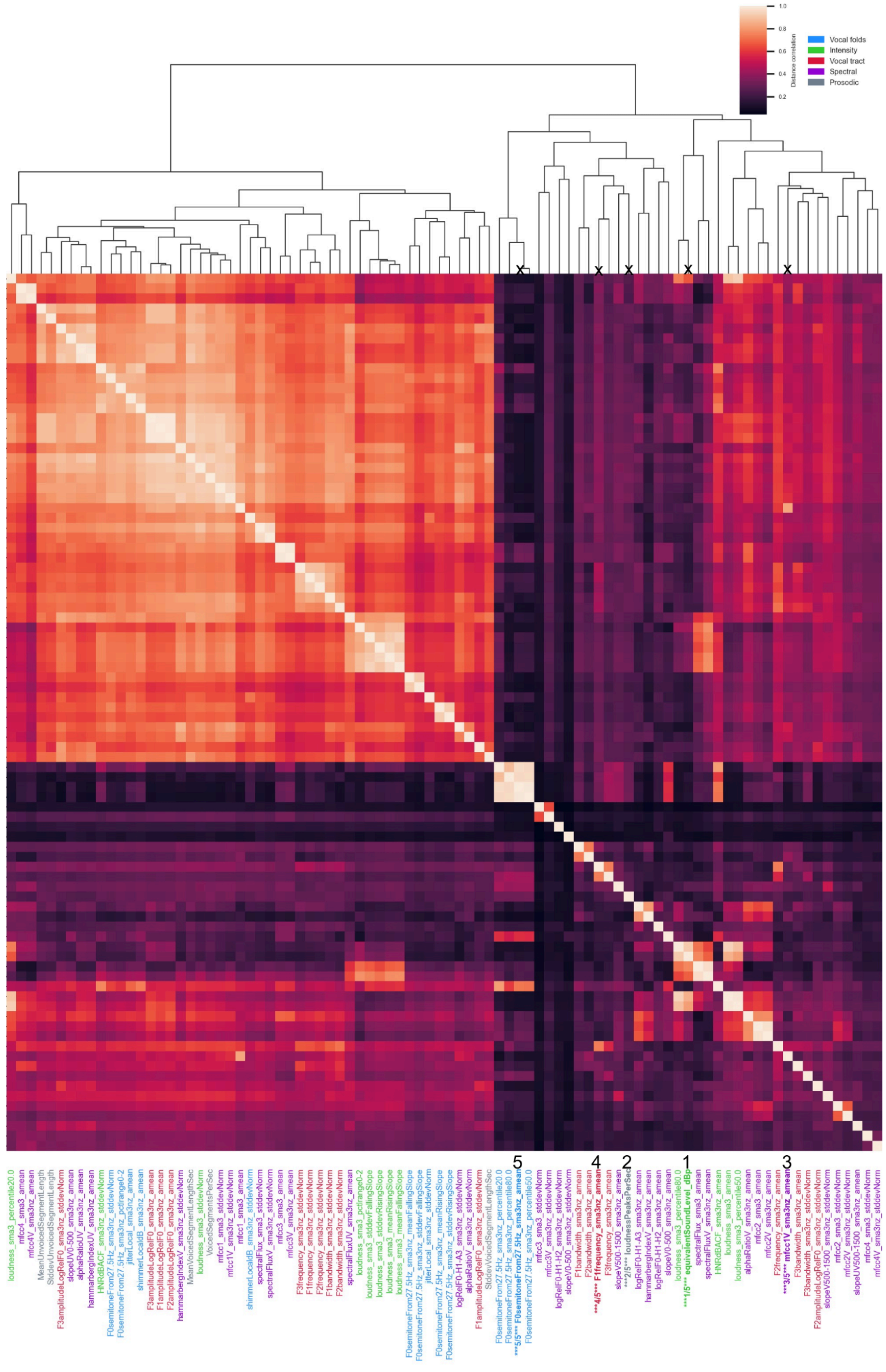

Figure K. Controls, reading+vowel tasks: Visualization of features with shared information using pairwise distance correlation across the 88 features. Squares are clusters of redundant features.

Performance with and without redundant features

While removing redundant features is important for explainability, it should not be at the expense of predictive performance. Therefore, we trained and evaluated models and progressively removed redundant features to observe how performance dropped with fewer and fewer features. For each data type (reading, vowel, reading+vowel), through visual inspection of Figure L, we chose the smaller feature set size that had similar performance to the full feature set size of 88 features: 39 features for reading, 13 features for vowel, and 19 features for reading+vowel.

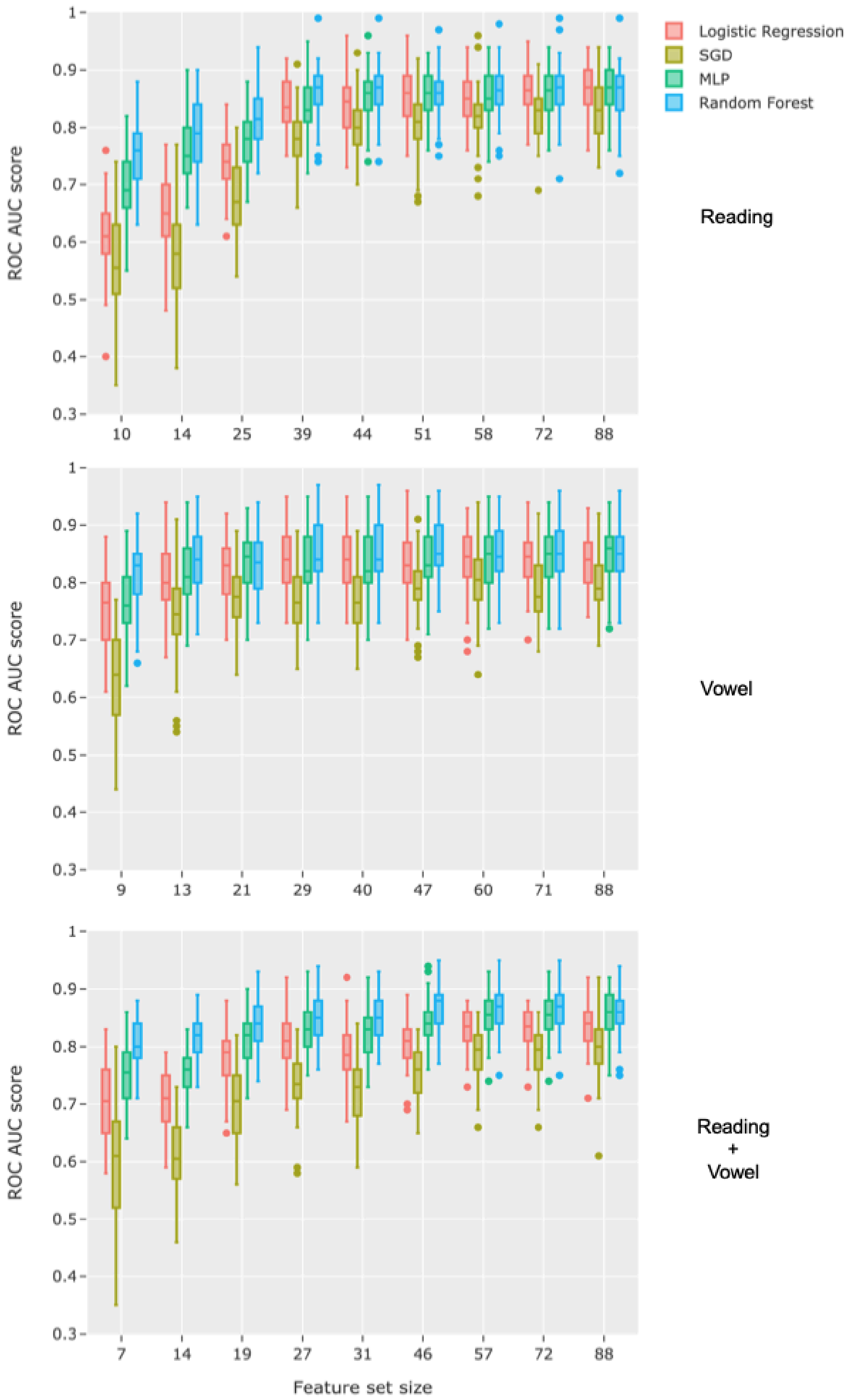

Figure L. Performance as a function of feature set size using Independence Factor method for reducing feature redundancy. The feature sets remove features with distance correlation ≥ 0.2 up to 1.0 (i.e., keeping all features) in increments of 0.1.

Feature Selection

To make sure information from the test sets is not having a strong influence on feature selection, we tested feature selection on 50 random train sets (80% of samples to match how models were trained) to make sure similar features were selected through this nested approach. If feature selection is relatively consistent across samples, removing features on the entire dataset should not be overfitting and is preferred for the explainability analysis to compare the same features. As seen in Table B, all or most of the features used by selecting on the data set were also the most common across 50 splits and were selected in 91%, 83% and 76% of splits for reading, vowel and reading+vowel, respectively. Therefore, similar features are selected using both methods, but selecting on the entire dataset is preferred for explainability purposes (i.e., to rank the same features by their importance across all bootstrapping splits).

Selection using		Reading	Vowel	Reading+Vowel
Entire dataset	Optimal threshold and selected features	0.5	0.3	0.4
	Selected features	39	13	19
50 bootstrap train sets	Selected features (mean [95% CI])	35.8 [34–38]	12.3 [11–14]	17.9 [16–20]
	Match between both methods (entire dataset / most common across 50 train sets)	39/39	12/13	16/19
	Selected in percentage of runs	91%	83%	76%

Table B. Comparison of selecting features on the entire dataset (useful for explainability) versus selecting on 50 bootstrap (80–20) train splits. Original total features are 88. CI = Confidence Interval.

Performance removing participants that used other recording system

Given 24 patients were recorded using an iPad, we trained models without their samples to make sure these differences in recordings were not driving performance. 66, 72, and 138 samples were removed from the reading, vowel, and reading+vowel datasets, respectively. Given the observed performance drop can also be due to removing training samples, the drop is not large enough to suspect that differences in recording are driving performance when using the full datasets (see Supplementary Table C).

	Features	LogisticRegression	MLP	RandomForest	SGD
Reading	88	.82 (.71–.87; .50)	.82 (.73–.88; .51)	.80 (.72–.88; .53)	.79 (.66–.87; .50)
Vowel	88	.78 (.71–.89; .50)	.79 (.68–.90; .54)	.81 (.73–.90; .52)	.74 (.60–.85; .45)
Reading+Vowel	88	.79 (.70–.87; .50)	.81 (.74–.88; .52)	.81 (.73–.88; .52)	.77 (.67–.84; .50)

Table C. Performance of models without 24 patients recorded on iPad. Median ROC AUC score from 50 bootstrapping splits (90% confidence interval; median score of null model). The control group represents 60% of the training samples. MLP: Multi-Layer Perceptron; SGD: Stochastic Gradient Descent Classifier.

Furthermore, we tested how well a model trained on all participants except those using the iPad and tested on the 24 UVFP patients that used the iPad (see Table D) to assess generalizability of the model to different recording settings. However, since the iPad recordings were all patients we can therefore only measure false negative rate but not ROC AUC. We used only the controls matched in age and sex to the remaining UVFP patients for training the models to maintain a balanced dataset (i.e., 53 UVFP patients and 53 matched controls).

	Features	LogisticRegression	MLP	RandomForest	SGD
Reading	88	0.08	0.26	0.09	0.39
Vowel	88	0.1	0.11	0.36	0.12
Reading+Vowel	88	0.12	0.2	0.12	0.12

Table D. False negative rate (FNR) of training on one recording device and testing on 24 UVFP patients that used iPad. FNR is generally quite low. Performance can also be influenced by having a smaller training set in order to balance the classes.

Biased features

We identified features that are biased (differ between groups not due to the intrinsic nature of UVFP, equivalentSoundLevel_dBp, and the other intensity-related features it is strongly associated with: loudness_sma3_amean, loudness_sma3_stddevNorm, loudness_sma3_percentile20.0, loudness_sma3_percentile50.0, loudness_sma3_percentile80.0, loudness_sma3_pctlrange0-2, loudness_sma3_meanRisingSlope, loudness_sma3_stddevRisingSlope, loudness_sma3_meanFallingSlope, loudness_sma3_stddevFallingSlope, loudnessPeaksPerSec, equivalentSoundLevel_dBp, HNRdBACF_sma3nz_amean, and HNRdBACF_sma3nz_stddevNorm.

We correlated these intensity features with all other features and removed the 43 features that had a distance correlation > 0.3 as seen in Table E. We correlated the audio duration with all other features and removed the 44 features that had a distance correlation > 0.3 as seen in Table E.

**Features associated with intensity features**	**dcor**
spectralFluxV_sma3nz_amean	0.9
spectralFlux_sma3_amean	0.89
F0semitoneFrom27.5Hz_sma3nz_amean	0.88
F0semitoneFrom27.5Hz_sma3nz_percentile20.0	0.88
F0semitoneFrom27.5Hz_sma3nz_percentile50.0	0.86
F0semitoneFrom27.5Hz_sma3nz_percentile80.0	0.82
spectralFluxUV_sma3nz_amean	0.78
slopeUV500-1500_sma3nz_amean	0.7
F0semitoneFrom27.5Hz_sma3nz_stddevNorm	0.57

slopeV500-1500_sma3nz_amean	0.57
spectralFlux_sma3_stddevNorm	0.54
shimmerLocaldB_sma3nz_amean	0.53
shimmerLocaldB_sma3nz_stddevNorm	0.48
slopeV0-500_sma3nz_amean	0.47
F3frequency_sma3nz_amean	0.46
VoicedSegmentsPerSec	0.45
F0semitoneFrom27.5Hz_sma3nz_pctlrange0-2	0.44
F1bandwidth_sma3nz_amean	0.43
F2frequency_sma3nz_amean	0.43
MeanUnvoicedSegmentLength	0.42
F2amplitudeLogRelF0_sma3nz_amean	0.4
F3amplitudeLogRelF0_sma3nz_amean	0.4
jitterLocal_sma3nz_amean	0.39
F2amplitudeLogRelF0_sma3nz_stddevNorm	0.39
F3amplitudeLogRelF0_sma3nz_stddevNorm	0.39
F1frequency_sma3nz_amean	0.38
jitterLocal_sma3nz_stddevNorm	0.38
MeanVoicedSegmentLengthSec	0.37
F1frequency_sma3nz_stddevNorm	0.37
mfcc1_sma3_amean	0.37
F1amplitudeLogRelF0_sma3nz_amean	0.36
spectralFluxV_sma3nz_stddevNorm	0.35
F1amplitudeLogRelF0_sma3nz_stddevNorm	0.35
F2frequency_sma3nz_stddevNorm	0.35
StddevUnvoicedSegmentLength	0.34
mfcc4_sma3_amean	0.34
alphaRatioV_sma3nz_stddevNorm	0.33
mfcc4V_sma3nz_amean	0.33

hammarbergIndexV_sma3nz_stddevNorm	0.32
F0semitoneFrom27.5Hz_sma3nz_meanFallingSlope	0.32
F3bandwidth_sma3nz_stddevNorm	0.31
F3frequency_sma3nz_stddevNorm	0.31
F2bandwidth_sma3nz_amean	0.31
mfcc2_sma3_amean	0.3

Table E. Features with distance correlation (dcor) > 0.3 with biased intensity-related features.

In Table F, we report the distance correlations between all 88 features with audio duration to observe which of the features is associated with the observed systematic bias between groups (Figure 2). Among the highest associations, we find the longer the recording the higher the intensity/loudness variables, which is expected given all control recordings are 3.5 s while the only recordings above this duration are all UVFP. Our clinician ratings confirmed that UVFP have higher background noise and perceived louder speech due to recording biases that we've discussed. Therefore, being a UVFP patient is likely confounding the association between duration and loudness (likely causing both to be higher and therefore to be associated). Therefore, all distance correlations with audio duration could be spurious or partially biased because they can also be explained by differences between UVFP and controls. Therefore, we do not think these distance correlations with audio duration should be overinterpreted for potential ways duration could be affecting features beyond UVFP-control differences. Of course, after trimming audio duration so that all samples are the same length, there is no variation in duration to compute these correlations; this specific bias is mitigated.

**Features associated with audio duration**	**dcor**
equivalentSoundLevel_dBp	0.76
spectralFlux_sma3_amean	0.72
loudness_sma3_stddevRisingSlope	0.71
loudness_sma3_amean	0.7
spectralFluxV_sma3nz_amean	0.7
spectralFluxUV_sma3nz_amean	0.7
loudness_sma3_percentile80.0	0.69
slopeUV500-1500_sma3nz_amean	0.67
loudness_sma3_percentile50.0	0.67

loudness_sma3_meanRisingSlope	0.65
loudness_sma3_percentile20.0	0.65
loudness_sma3_pctlrange0-2	0.64
loudness_sma3_stddevFallingSlope	0.64
loudness_sma3_meanFallingSlope	0.6
F0semitoneFrom27.5Hz_sma3nz_pctlrange0-2	0.54
F0semitoneFrom27.5Hz_sma3nz_stddevNorm	0.53
jitterLocal_sma3nz_amean	0.5
slopeV500-1500_sma3nz_amean	0.46
loudnessPeaksPerSec	0.46
F0semitoneFrom27.5Hz_sma3nz_percentile20.0	0.45
hammarbergIndexUV_sma3nz_amean	0.44
alphaRatioUV_sma3nz_amean	0.43
HNRdBACF_sma3nz_stddevNorm	0.42
F0semitoneFrom27.5Hz_sma3nz_meanFallingSlope	0.42
logRelF0-H1-A3_sma3nz_stddevNorm	0.42
HNRdBACF_sma3nz_amean	0.41
F0semitoneFrom27.5Hz_sma3nz_stddevRisingSlope	0.4
spectralFluxV_sma3nz_stddevNorm	0.39
F0semitoneFrom27.5Hz_sma3nz_stddevFallingSlope	0.37
F0semitoneFrom27.5Hz_sma3nz_meanRisingSlope	0.36
spectralFlux_sma3_stddevNorm	0.34
shimmerLocaldB_sma3nz_amean	0.33
StddevVoicedSegmentLengthSec	0.32
shimmerLocaldB_sma3nz_stddevNorm	0.31

Table F. Features with distance correlation (dcor) > 0.3 with biased audio duration.
